# Study protocol for a three-armed randomized controlled trial to assess whether house screening can reduce exposure to malaria vectors and reduce malaria transmission in The Gambia

**DOI:** 10.1186/1745-6215-9-33

**Published:** 2008-06-06

**Authors:** Matthew J Kirby, Paul J Milligan, David J Conway, Steve W Lindsay

**Affiliations:** 1 Durham University, Science Laboratories, South Road, Durham, DH1 3LE, UK; 2 Medical Research Council Laboratories P.O. Box 273, Banjul, The Gambia; 3 London School of Hygiene and Tropical Medicine, Keppel Street, London WC1E 7HT, UK

## Abstract

**Background:**

Mosquito-proofing homes was one of the principal methods of environmental management in the early 1900s. House screening provides protection against malaria by reducing exposure to malaria parasites and has the added benefit of protecting everyone sleeping in the house, avoiding issues of inequity within the household. The aim of this study is to determine whether house screening protects people against malaria in Africa. It is hoped that this study will mark the beginning of a series of trials assessing a range of environmental interventions for malaria control in Africa.

**Design:**

A 3-armed randomised-controlled trial will be conducted in and around Farafenni town in The Gambia, West Africa, to assess whether screening windows, doors and closing eaves or installing netting ceilings in local houses can substantially reduce malaria transmission and anaemia compared to homes with no screening. Eligible houses will be sorted and stratified by location and the number of children in each house, then randomly allocated to the interventions in blocks of 5 houses (2 with full screening, 2 with screened ceilings and 1 control house without screening). Risk of malaria transmission will be assessed in each house by routine collections of mosquitoes using light traps and an anaemia prevalence study in children at the end of the main transmission period.

**Discussion:**

Practical issues concerning intervention implementation, as well as the potential benefits and risks of the study, are discussed.

**Trial Registration:**

ISRCTN51184253 – Screening-homes to prevent malaria

## Background

Malaria remains one of the greatest childhood killers in the world[1] and is a substantial obstacle to social and economic development[2]. We know from historical accounts that in the early 1900s malaria was controlled using environmental management (EM) for vector control in many parts of the tropics [3-5]. EM was effective and sustainable in controlling and eradicating malaria, but was forgotten during the DDT campaigns of the 1950s and 1960s. Today most control tools rely exclusively on chemicals (anti-malarial drugs and insecticides), not on environmental modifications, nor on strengthened social systems to perform effective environmental manipulation. Whilst drugs and insecticides are extremely effective weapons, their initial promise has been compromised by the development of resistance [6-10] and growing concerns about long-term environmental impacts[11]. It is therefore of considerable strategic importance to reduce our dependency on antimalarials and insecticides by developing effective and sustainable methods of control. EM could help achieve this and provides new opportunities for the control of malaria that could be incorporated into integrated disease control programmes, providing a more effective, environmentally friendly and sustainable approach to malaria control.

**Figure 1:**
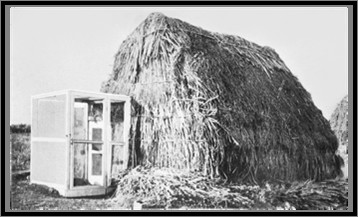
A screened entrance to a thatched house in rural Italy in 1900, in an experiment where screening reduced malaria by 96% [20]. This illustrates that even extremely poor housing can be protected from mosquitoes.

Mosquito-proofing homes was one of the principal methods of EM in the early 1900s (Fig 1) and it should be effective against malaria in Africa since the majority of people receive infective bites at night indoors.

The literature review of Lindsay *et *al. provides compelling evidence that house screening has been associated with protection against malaria transmission, infection and morbidity[12]. Furthermore, a pilot study conducted in The Gambia using experimental huts demonstrated that netting ceilings alone can reduce exposure to malaria vectors by 80%[13]. In this situation, most mosquitoes entered the huts through the open eaves and were effectively trapped in the roof space. Further studies in actual village houses are needed to confirm these findings and to assess the acceptability of the intervention to house-holders.

## Methods

### Study area, house- and participant eligibility

The study area is situated approximately 170 km from the mouth of the Gambia River and covers 70 km^2 ^of the North Bank Division in The Gambia, an area of open Sudan savanna vegetation. The climate consists of a single rainy season from June to October followed by a long dry season. Specifically, the study area comprises 500 houses; 46 in 11 residential blocks in Farafenni town (UTM coordinates: 1500200N, 435500E) and 454 in 25 villages located within 15 km of the town. Farafenni itself is a market town with a population of over 20,000 inhabitants, situated less than 5 km from the river and 2 km south of the border with Senegal. Houses in both the town and surrounding villages are usually arranged in familial compounds demarcated by a fence or wall, though there are some in which the houses are rented by unrelated family groups. Compounds in Farafenni contain typically 1–4 houses and in the villages 4–6 houses, but sometimes as many as 20. Houses must meet 6 eligibility criteria in order to be selected: (1) they must be single storey buildings, (2) have open eaves, (3) with no more than four rooms, (4) have no existing ceilings, (5) have no screening and (6) at least one child, aged 6 months to 10 years, must sleep in the house at night. Most of the houses to choose from are part of the Farafenni demographic surveillance system (FDSS) that incorporates 46 residential blocks in the town and 23 surrounding villages, also defined as individual blocks within the FDSS.

The study area population comprises 7852 people dominated by three ethnic groups; Mandinka (28%), Wollof (38%) and Fula (27%). There are roughly equal numbers of men (53%) and women. The study aims to recruit 1250 children aged 6 months to 10 years, as this is the age group most affected by the clinical manifestations of malaria. No distinctions are made regarding gender or ethnic group. Moreover, as the target population will in reality be all children occupying study houses, and in order for the results from this study to be broadly applicable, no distinctions are made in terms of medical condition or physical health.

### Sensitization

Village meetings will be held to explain to the villagers the purpose of the study and the benefit they will receive from participation in the trial. Each meeting will end with an opportunity for asking and answering questions. Informed verbal and written consent is sought from the parents or guardians of children who fulfil the entry criteria for the study.

### Design

In each of 2 intervention years we will erect; full screening on the door(s) and windows and close the eaves with a mixture of sand, rubble and cement (as is normal local practice) in 100 houses and install screened ceilings in a further 100 houses. 50 houses without any screening will serve as a control group. Thus we will enroll a total of 500 houses over the 2 year study. Screening will be installed prior to the rainy season of each year (January to May). All rooms in the house will be screened, not just the target bedroom(s).

Exposure to mosquitoes will be measured by routine surveillance with light traps, fitted near a participant sleeping under an untreated bednet. All children sleeping in eligible houses will sleep under an untreated bednet and their exposure to malaria parasites estimated by (1) haemoglobin density (Hb), (2) percentage of children with anaemia (<8 g/dL), (3) percentage of children with severe anaemia (<5 g/dL), (4) parasite prevalence and (5) high parasite prevalence (> = 5000 parasites/μl), as determined by a cross-sectional survey at the end of the transmission season. At the end of the trial the participants in the control group will be able to choose to have their houses screened, and participants in either of the screening groups will be able to choose to keep that screening, have it removed, or have it changed to the other type of screening. In addition, insecticide-treated nets (ITN) will be provided to all study subjects at the end of the trial.

### Randomisation

Eligible houses will be sorted and stratified by (1) rural (village) or urban (Farafenni) location, (2) block and (3) the number of children in each house. The randomization list will be generated by PM in blocks of 5 (2 full screening interventions: 2 ceiling screening interventions 1: control) using Stata 7(StataCorp., College Station, TX, USA). The randomization should give a reasonable balance between groups in the roof type, ITN use, the number of malaria vectors (*Anopheles gambiae s.l*.) caught from the houses in a pilot study [14], and the number of doors and windows. Ideally we would have preferred to hold a raffle to allocate the intervention and control treatments to houses, so that the participants could witness the evenhandedness of the randomization. However, because the exact number of houses within each randomization block is known, it would not be possible to conceal the identity of the last allocation to be drawn in each block. If the last ticket were known to be a control ticket, there is the possibility of it not being drawn and the participant withdrawing from the study, resulting in a potential source of bias. Participants will therefore be enrolled into their respective groups by the project field staff.

### Interventions

#### a) Full Screening arm

**Figure 2:**
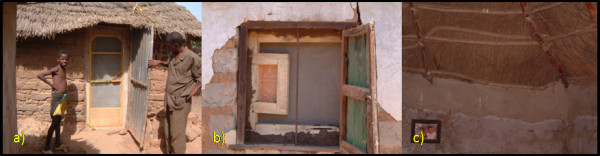
Installation of full screening intervention: a) screened door, b) screened window, c) closed eaves.

Door frames will be made from 50 × 50 mm softwood. The doors themselves will be constructed from 15 × 30 mm softwood, strengthened at the corners. Sheets of white PVC-coated fibreglass netting (Vestergaard Frandsen group, Denmark) will be stretched and stapled over the doors (Fig 2a). All doors will open inwards and will be hung on the inside of any existing doors, unless prevented from doing so by an immovable obstruction. Handles and push bars will be fitted on both sides of the door and a catch fixed on the inside to hold the door tight in the frame when shut. Elastic cord will be attached to the outside of the door to pull the door closed when not in use. Windows will be made in a similar fashion to the doors. If the occupants do not need to open the window the netting will be stapled straight onto the 50 × 50 mm frame. If there is already a window in place then the screened window will be installed to the inside of the existing window, and a sliding plywood panel fitted to enable access to the outer window (Fig 2b). Eaves will be closed with a mixture of sand, rubble and cement (Fig 2c).

#### b) Netting ceiling arm

**Figure 3:**
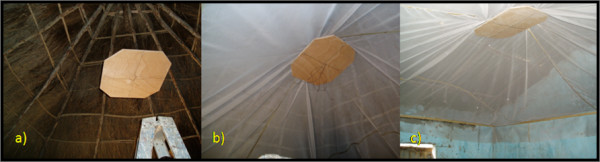
Installation of netting ceiling intervention: a) assessing rose height, b) installing ceiling and rose, c) installing battens.

Sheets of 2.4 m wide white PVC-coated fibreglass netting (Vestergaard Frandsen group, Denmark) will be cut to fit the room (leaving at least 50 cm overhang on all sides), over-lapped and stitched together with ribbon by a local tailor. The netting will be centered over a plywood ceiling rose which is tied by wire to a ceiling beam in the centre of the room (Fig 3a,b). The overhang of the netting will be tucked behind 10 × 40 mm softwood battens which will then be screwed securely to the top of the walls (Fig 3c).

#### c) Control arm

Control houses receive no screening of any type and the eaves remain open.

### Mosquito collection

Each study house will be sampled biweekly during the rainy season. A CDC miniature light trap (Model 512, John W. Hock Co., Gainesville, FL) will be positioned 1 m above the ground within 1–2 m of the foot end of a bed protected with a new untreated bednet provided on that night only. If the trapping room contains multiple beds, then the other room occupants will be encouraged to use their existing bednets. If they have none, additional new bednets will be provided for that night. Light traps will be operated from 19:00 to 07:00 h the following morning. Mosquitoes will be killed by freezing at -25°C for 2 hours and identified using morphological criteria. 1% of the *An. gambiae s.l*. complex caught in the traps will be identified to species by PCR analysis

### Clinical data collection and patient treatment

All children older than 6 months and under the age of 10 years living in eligible houses in the study blocks will be will be issued with an identity (ID) card, which they will present for their correct identification in November at the time of finger-pricking. Axillary temperature will be measured on arrival at the clinical station. A rapid dipstick test (ICT malaria P.f Cassette Test, ICT Diagnostics, South Africa) will be done for any child presenting with temperature ≥ 37.5°C and/or history of fever in the last 48 h. A blood sample will be taken from all children to measure haemoglobin using a portable β-haemoglobin photometer (Hemocue^®^, Ängelholm, Sweden).

In addition, using blood from the same finger-prick, the nurse will make a thin and thick film for confirmation and quantification of malaria parasites. Slides will be numbered with the date of finger-pricking and the subject's randomly generated ID number, and transported to MRC Laboratories Field Station, at Farafenni. Thick blood films will be stained with Giemsa and examined under 1000-fold magnification. Parasite counts will be counted per high power field and 50 fields counted before a slide is declared negative. Parasite density per μL was calculated assuming a blood volume per HPF of 0.002 μL. Thin films will be stained in the field with Field's stain of water-based Romanovsky. *Plasmodium *species will be identified from thin films.

Children with Hb < 8 g/dL will be classified as anaemic and given iron supplementation. Chloroquine and Fansidar will be given to any child ICT-positive, and also to children who were ICT-negative or not tested but were subsequently blood-slide positive. The parents of any child treated for malaria will be asked to bring their child to the nearest Maternal and Child Health clinic if the child is sick and does not recover within 48 h. Children with Hb <5 g/dL will be taken to the Farafenni hospital and followed up for two weeks after fingerpricking.

Clinical data will be recorded initially on the appropriate forms, double entered into a computer by two separate data entry clerks using Microsoft Access 2000, verified and errors amended where appropriate. The original clinical data will be kept in a locked cabinet and the key kept by the local investigator. The digitized dataset will be kept in a server, password protected and only accessed by the data manager and PI at the MRC Laboratories in Farafenni, and the data manager at the central MRC site in Fajara. Data will be stored for at least 10 years.

### Sample Size Rationale

#### a) Entomological

It is considered that a reduction of at least 50% in mosquito catch is needed if the intervention is to be considered of value as a practical public health intervention. In pilot studies of the ceiling intervention in experimental huts[13], the reduction observed was 79% (95% CI 40–94%). A 50% reduction was considered achievable in an operational setting. Based on preliminary data a geometric mean catch of 15 female *Anopheles gambiae *s.l./trap/night was anticipated in the control group, and a catch of 7.5 or less in intervention groups. It was considered that interventions which differ in terms of mean catch by 2.5 mosquitoes/trap/night or less would be considered equally effective. The trial was therefore planned to have 90% power to detect a 50% reduction in mosquitoes between the control group and either intervention group (i.e. from 15 to 7.5 mosquitoes/trap/night).

In addition we want to be able to rule out the possibility of important differences between the two interventions. Providing both interventions reduce mosquito biting by more than half we think the choice of intervention will be largely based around whether users like the screening and the cost of the intervention. However, assuming both interventions are liked equally well and they are of a similar cost, we consider that a difference in protection smaller than 50% in one arm and 67% in another would not be of interest to health providers since the difference would need to be greater in order to make an appreciable change to the clinical pattern of malaria in a setting. In Farafenni town we anticipated that, on average, people would receive around one infective bite each year. Studies in Tanzania have shown that a difference of 0.5 (50% reduction) and 0.33 (67% reduction) would, for example, have little impact on malaria prevalence (Bǿdker *et al*., unpublished data).

We have based our sample size calculations on discriminating between a mean of 7.5 (50% reduction) and 5.0 (67% reduction) mosquitoes/trap/night in the two intervention groups. Assuming a SD of log(e) catch of 1.2, 181 houses per intervention group would be needed for a 2-group 1-sided t-test to have 90% power to reject the null hypothesis that the difference between the group means is 2.5 or more, with an alpha-level of 0.025 and assuming the expected difference between the means is zero.

Thus in a three arm trial we required 200 houses randomized in each intervention group and 100 in the control arm, allowing for 10% of houses to be excluded from the primary analysis due to withdrawal or non-adherence to protocol.

#### b) Clinical

Our aim was to have 90% power to detect a difference in mean haemoglobin of 0.5 g/dl between the two intervention arms and the control arm in November, using a significance level of 2.5% for each of the two comparisons of intervention with control. In a recent trial in The Gambia the SD was 1.7 g/dl. A difference of 0.5 g/dl is 0.3SD. The interclass correlation (ICC) for haemoglobin in another trial in Senegal varied from 0.04 to 0.08. Assuming a similar ICC in the present trial the number of children required to detect (with 90% power) a difference of 0.5 g/dl is 166 in the control arm and 332 in each intervention arm.

### Analysis

#### a) Trial profile

All children in the study will be described in a flow chart and/or the text to indicate, for each arm of the trial, the number of children recruited, the number lost to follow up and reasons for loss to follow up, and the number sampled in November.

The trial profile will also show the number of houses considered for enrolment, the number excluded from the study with reasons for exclusion, the number randomized in each group, and the number withdrawn with reasons for withdrawal.

#### b) Intention to treat (ITT) and according to protocol (ATP) analysis sets

The analysis of primary and secondary endpoints will be considered two ways. Firstly we will adopt an 'intention to treat' approach where the analysis is carried out using all households that are randomized and for which there are some outcome data. At enrolment of the house, a list of children who normally sleep in the house will be made and updated at the beginning of the rainy season to include newborns and record any changes in occupancy. At blood sampling in November, any children absent will be listed with the reason they were missing. The ITT study population for the anaemia endpoints is all children included in the updated list. Analysis will be limited to those who were sampled for Hb and parasitology in November.

Secondly, we will adopt an 'according to protocol' approach where the analysis is carried out using all houses and study subjects in the control group, but excluding all houses from either intervention group (and study children that slept there) that have screening scored as 'badly damaged' in a durability study conducted 6 months post screening installation. The definition of 'badly damaged' screens will be determined separately for homes in the two treatment arms. In full screened houses damage will equate to 5 or more holes in the screening and/or doors not closing tightly. In screened ceiling houses damage will equate to 5 or more holes in the screening and/or where the netting has come away from the battens that secure the screening to the walls.

ITT and ATP analyses will include stratification by year, by rural/urban, and by block, which were stratification factors in the randomization. There will be no adjustment for the number of children in each trial arm even though this was a stratification factor in the randomization. We stratified to ensure similar numbers in each trial arm for Hb sampling, but the number of children in the house is not expected to relate to any of the clinical outcome measures.

#### c) Data exclusions

Data exclusions will be tabulated for each trial arm. The following data sets will be excluded from the analysis: (1) light trap collections on nights when the light trap was not working, (2) houses destroyed or vacated by residents, (3) houses for which the occupier withdrew consent, (4) evaporimeter readings greater than 30 mm.

#### d) Primary analysis of variables

##### Entomological data

Comparisons of mean number of mosquitoes/house between intervention groups and the control group will be made using ANOVA on the log mean catch for each house. The mean will be calculated firstly as the Williams mean (geometric mean after adding 1 to all values), and in a secondary analysis, as the arithmetic mean. To allow for the 2 comparisons being made (between the control arm and each of the two interventions), P-values less than 0.025 will be considered significant and 97.5% confidence intervals will be calculated. For the comparison between the two intervention groups, we will estimate the 95% confidence interval for the ratio of the mean catch per house. If the upper limit of this interval corresponds to a difference in catch of 2.5 or less, the ceiling intervention will be considered as effective as full screening.

Estimates of entomological inoculation rate (EIR) will be generated by multiplying the mean number of mosquitoes/adult/house/night by the sporozoite rate (adjusted for area and year) and multiplying this by the number of nights during each year. The sporozoite rate, with a 95% confidence interval, will be estimated for each arm of the trial based on the number of positive and negative pools and the number of individuals per pool, and a confidence interval for the difference between trial arms will be estimated for each pairwise comparison.

##### Clinical data

For each house, the mean Hb will be calculated and these means compared between each intervention group and the control group using ANOVA.

#### e) Secondary analysis

##### Entomological data

The secondary analysis will impute missing mosquito catch data for nights when individual houses could not be sampled. The analysis will be done adjusting for the same variables as the primary analysis but including the following covariates that have been shown to be associated with mosquito catch size[14]: (1) presence of horse(s) tethered near the house at night, (2) churai burnt in the trapping room at night, (3) number of people sleeping in the trapping room at night, (4) wall material (mud brick or concrete).

Night-to-night variation in covariates 1, 2 and 3 can be accounted for as these will be recorded on every visit to each house. Covariate 4 will obviously remain constant throughout the trapping period for each house. We will also include the presence of one or more ITNs in the house, and socioeconomic status, in the list of covariates. Socioeconomic status will be determined by principal component analysis (PCA) from a list of household assets.

Generalised estimating equations (GEE) will be used to estimate treatment effect, adjusting for covariates. Differences in house entry by malaria mosquitoes experienced in the three groups will be analysed based on count data, using a generalised estimating equation to account for the correlation between the longitudinal observations in the same house comparing the number of mosquitoes caught indoors in each house in each intervention group with the control group.

##### Clinical data

The secondary analysis will be done adjusting for the same variables as the primary analysis but including all the covariates listed in the entomological analysis, with three changes: (1) whether the study subject reports sleeping under an ITN (rather than whether someone in the trapping room uses an ITN as in the entomological analysis), (2) whether the net was in good condition or not. A net in good condition is one which is long enough to be tucked under the mattress, is not torn or otherwise damaged, and had no more than 5 small holes (finger-width, approximate diameter ≤ 2 cm [15], (3) child's age. Interactions with age will be investigated using age groups: under 2 years old, 2–5 years, and 5–10 years old.

For the clinical variables (haemoglobin, anaemia, severe anaemia, parasitaemia, high density parasitaemia and parasite density in infected children), generalized estimating equations (GEE) will be used to estimate the odds ratios between each intervention group and the control group.

Comparisons of clinical variables (haemoglobin, anaemia, severe anaemia, parasitaemia, high density parasitaemia and parasite density in infected children) between intervention groups will be reported as the difference in risk or the difference between means, with 95% confidence intervals.

##### Ethical Approval

Ethical approval for this study was given by the Gambian Government and Medical Research Council Laboratories Joint Ethical Committee and the Ethics Advisory Committee of Durham University.

## Discussion

This is a proof-of-concept trial to determine whether screening can reduce malaria transmission. It is designed to measure whether either full screening or screened ceilings can reduce the exposure to *An. gambiae s.l*., the principal vectors of malaria in Africa, and whether the interventions can reduce anaemia in children.

Anaemia is a clinically relevant measure of malaria in children. Typically in areas of intense seasonal transmission like The Gambia it is measured in the same children at the beginning and end of the rainy season [16-19]. However, we choose not to measure anaemia at the start of the rainy season because anaemic children would have to be treated, interfering with the natural history of anaemia in this community. Whilst we cannot be completely confident that the proportion of anaemic children will be the same in each arm of the trial, randomization should result in comparable groups. The only other foreseeable problem is the difficulty of locating the study participants. The local population is highly mobile, particularly the families of the herdsmen of the Fula ethnic group. Additionally the children of all ethnic groups are often sent away for schooling or weaning; this obviously presents difficulties in associating child health with one particular house.

As far as possible, the interventions themselves will be made using locally available materials. Although we will be using high quality netting for screening, locally available netting, although of inferior quality, is available in Farafenni town. All timber, screws, nails and elastic cords will be purchased locally and the interventions installed by local carpenters. We aim is to achieve a standard screening design within each intervention arm. However, wall height poses a problem for installing ceilings. In houses with high walls the netting will be stretched horizontally across the room as this is the easiest installation technique. In houses with low walls the ceiling must be pulled up from the central rose into the roof space to allow enough room at head height. In the full screening intervention arm, installing door frames into houses with narrow entrances further restricts access. The best way to obviate this will be to widen the entrance, if the participants agree to this. Because of the large number of intervention houses and the necessity to repeatedly sample for mosquitoes during the rainy season it was felt prudent to carry out the study over 2 successive rainy seasons so as not to overburden the study team.

There are no apparent risks to the safety of individuals in this trial, although finger-pricking is mildly uncomfortable and the rooms may be stuffier when screened. However, there are a number of benefits for participants in this trial. Prompt treatment for children with malaria and anaemia will be provided for children at the end of each rainy season. New untreated bednets will be provided when mosquito sampling and ITNs given to all participants at the end of the trial. We anticipate that the interventions will be protective and that the 'no intervention' control group will benefit by being given netting ceilings or full screening at the end of the study period.

The findings from this study will enable us to evaluate a range of different methods for screening homes against mosquitoes. By focusing on an assessment of reducing mosquito transmission in this study it will be possible to better plan a second trial in order to assess the impact of screening on the incidence of malaria morbidity in children. Lessons learned from the trial will benefit the study community, the Gambian Medical and Health Department and other African Departments of Health. Although house screening needs to be tailored to local house designs, the general principles involved in this program should help inform malaria control in many other African countries and other parts of the tropics. The results of this program will therefore be of interest to malaria control programs, local administrations, industries with large labour forces and non-Governmental Organisations throughout sub-Saharan Africa. Moreover, there is growing international awareness amongst the research and development communities that EM needs to be re-evaluated in Africa today and projects such as this will help guide the development of sustainable methods for protecting the poor and vulnerable from malaria.

## Competing interests

All the PVC-coated fibreglass netting used by this project has been received gratis from Vestergaard-Frandsen group.

## Authors' contributions

MK designed the interventions, contributed to the design of clinical aspects of the protocol and analytical plan and drafted the manuscript, SWL conceived the study, its design and coordination and helped draft the manuscript and analytical plan, PM helped with sample size calculations and the analysis plan, DC helped design the study. All authors read and approved the final manuscript.
